# TGF-β Signaling Initiated in Dendritic Cells Instructs Suppressive Effects on Th17 Differentiation at the Site of Neuroinflammation

**DOI:** 10.1371/journal.pone.0102390

**Published:** 2014-07-29

**Authors:** Suzanne Speck, James Lim, Sagar Shelake, Marsel Matka, Jonathan Stoddard, Alexander Farr, Vijay Kuchroo, Yasmina Laouar

**Affiliations:** 1 Department of Microbiology and Immunology, University of Michigan School of Medicine, Ann Arbor, Michigan, United States of America; 2 Evegrande Center for Immunologic Diseases, Harvard Medical School and Brigham and Women's Hospital, Boston, Massachusetts, United States of America; University of Iowa Carver College of Medicine, United States of America

## Abstract

While the role of Transforming Growth Factor β (TGF-β) as an intrinsic pathway has been well established in driving *de novo* differentiation of Th17 cells, no study has directly assessed the capacity of TGF-β signaling initiated within dendritic cells (DCs) to regulate Th17 differentiation. The central finding of this study is the demonstration that Th17 cell fate during autoimmune inflammation is shaped by TGF-β extrinsic pathway via DCs. First, we provide evidence that TGF-β limits at the site of inflammation the differentiation of highly mature DCs as a means of restricting Th17 cell differentiation and controlling autoimmunity. Second, we demonstrate that TGF-β controls DC differentiation in the inflammatory site but not in the priming site. Third, we show that TGF-β controls DC numbers at a precursor level but not at a mature stage. While it is undisputable that TGF-β intrinsic pathway drives Th17 differentiation, our data provide the first evidence that TGF-β can restrict Th17 differentiation via DC suppression but such a control occurs in the site of inflammation, not at the site of priming. Such a demarcation of the role of TGF-β in DC lineage is unprecedented and holds serious implications vis-à-vis future DC-based therapeutic targets.

## Introduction

TGF-β is central to the evolution of host defense and protection from autoimmunity. This factor represents the prototypic member of a superfamily of structurally and functionally related peptides that affect many different cellular processes [1]. TGF-β was originally recognized for its pro-inflammatory properties, but identification of its powerful suppressive activities focused attention for the last decades on dissecting its mechanisms on immune inhibition [2]–[5]. Just as quickly as TGF-β-mediated regulation of regulatory T cells became evident [6], [7], a surprising finding that TGF-β induced differentiation of pro-inflammatory Th17 cells emphasized a broader ability in dictating inflammatory events [8]–[12]. Whereas the role of TGF-β as a T cell-intrinsic signal has been well established in Th17 differentiation, much remains to be discovered in DC-dependent Th17 differentiation in the complex milieu of inflammation.

The microenvironment established at the site of inflammation is highly dynamic, favoring abundant secretion of inflammatory mediators, massive recruitment of leukocytes, and *in situ* formation of immune cells that can adopt different functions, including regulatory and inflammatory roles [13]. A cardinal feature of DCs in the neuroinflammatory setting is their ability to promote Th17 differentiation, known to be responsible for the pathogenesis of multiple sclerosis (MS), and its animal-model experimental autoimmune encephalomyelitis (EAE) [14]–[17]. DCs of the inflamed CNS serve in the local reactivation of myelin-specific T cells [18], [19], initiate epitope spreading in relapsing diseases [20], and inflict tissue damage through the secretion of toxic factors such as reactive oxygen species and tumor necrosis factor [21]. However, while a large body of work has been devoted to identifying the precursor cells that generate these DCs and the soluble factors that promote their differentiation, factors that limit their differentiation still await identification.

TGF-β has recently emerged as a major component of the inflammatory milieu established in the CNS during EAE. Visualization of TGF-β activity by bioluminescence imaging conclusively demonstrated that the CNS, not the periphery, is the major site for TGF-β activity during EAE [22], [23]. This information is essential for our understanding of the function of TGF-β in DC-dependent Th17 differentiation because it indicates a selective role of this interplay at the site of inflammation (CNS), rather than at the site of priming (periphery). The collective results from studies of TGF-β pathway manipulation during EAE have proved to be conflicting, with opposing effects arguing for both protective and pathogenic roles: On one hand, the inhibition of TGF-β activator TSP-1 delays EAE [23], and treatment with a pharmacological inhibitor of TGF-β receptor I ameliorates the disease [24]. On the other hand, the systemic inhibition of TGF-β by blocking antibody worsens the disease [25], and systemic provision of recombinant TGF-β appears to protect against EAE [26]–[28]. Although conflicting, these studies provide evidence that TGF-β is critical during EAE. Thus, scrutinizing its function on a cell-type basis is required to elucidate its opposing effects.

In the present study, we used a DC-specific blockade of TGF-β signaling (CD11c^dnR^ mice) to scrutinize the role of TGF-β in the DC compartment during EAE. We previously showed that development of EAE results in severe disease in immunized CD11c^dnR^ mice and causes spontaneous EAE in crossed CD11c^dnR^Mog^TCR^ mice [29]. To distinguish the role of TGF-β on DCs versus NK cells in EAE, we used an adoptive-cell transfer strategy and showed that a lack of TGF-βR signaling in DCs, but not NK cells, is responsible for severe EAE in CD11c^dnR^ mice [29]. Using radiation-BM chimeras, we showed that a lack of TGF-βR signaling in conventional DCs, but not CNS-resident microglial cells, is responsible for severe EAE in CD11c^dnR^ mice [29]. However, in spite of clear evidence that DC-intrinsic TGF-β signaling is critical in disease severity, analysis of TGF-β-resistant DCs in the periphery yielded no clues. Here, we show an unprecedented demarcation of the role of TGF-β in DCs in the inflammatory (CNS) versus priming (periphery) sites with consequences on Th17 cell fate and disease severity.

## Materials and Methods

### Mice

B6.Cg-Tg(SBE/TK-luc)7Twc/J (SBE-Luc) were purchased from Jackson Laboratories (Bar Harbor, ME). CD11c^dnR^ transgenic mice were used under C57BL/6 background. Mog^TCR^ transgenic mice on C57BL/6 background were obtained from Dr. Kuchroo (Harvard Medical School, Boston, MA). Mice were bred in-house to generate CD11c^dnR^Mog^TCR^ double transgenic mice. All mice were bred and maintained in a specific pathogen-free barrier unit at the University of Michigan. All experiments followed guidelines of the University of Michigan Animal Care and Use Committee. Approval for use of mice was obtained from the University of Michigan according to criteria outlined in the *Guide for the Care and Use of Laboratory Animals* from the National Institutes of Health.

### EAE induction

EAE was elicited by subcutaneous (s.c.) tail-base injection of 50 µg of MOG35–55 peptide (35-MEVGWYRSPFSRVVHLYRNGK-55) (U-M Protemocis and Peptide synthesis core facility) in complete Freund adjuvant (CFA) containing 500 µg of heat-inactivated *Mycobacterium tuberculosis* (serotype H37RA) (DIFCO Labs). A dose of 200 ng of *B. pertussis* toxin (LIST Biological Labs, Inc.) was injected intra peritoneally (i.p.) on days 0 and 2. Mice were monitored daily for clinical signs of EAE and were scored as follows: 1, flaccid tail; 2, inability to right; 3, one hind limb paralysis; 4, paralysis of both hind limbs; and 5, moribund. Supplementary food and water were provided on the cage floor for disabled animals.

### Live mouse imaging

EAE was elicited in B6.Cg-Tg(SBE/TK-luc)7Twc/J (SBE-Luc) mice as described above, and live mice were imaged at different times during EAE development. To perform live-mouse imaging, mice were injected i.p. with 150 mg/kg D-luciferin (Xenogen) 10 min before imagining. During this time, mice were left in an anesthesia unit to ensure their immobility during imaging. Bioluminescence images were taken with the *In Vivo* Imaging System 100 (IVIS; Xenogen), and photons were integrated for 2 minutes and measured as photons per second per square centimeter per steradian. Bioluminescent images were superimposed onto gray-scale images that were taken before imaging. Superimposition and analysis of images were performed using LIVINGIMAGE software version 2.11 (Xenogen).

### Isolation of CNS-infiltrating leukocytes

To isolate CNS-infiltrating leukocytes, mice were perfused intracardially with ice-cold PBS. Brains and spinal cords were harvested and cell suspensions were subjected to Percoll (Pharmacia) gradient, according to standard procedure. Mononuclear cells containing CNS-infiltrating leukocytes were collected from the 37∶70% interface. Cells were washed twice and counted.

### Dendritic cell derivation and activation

Bone-marrow cells were cultured for 6 days in RPMI-1640 supplemented with 10% FBS (Hyclone), 1X Penicillin Streptomycin Glutamine solution (Gibco), and 1∶3000 GM-CSF supernatant (U-M Hybridoma core facility). GM-CSF-supplemented media was changed every second day (days 0, 2, 4, and 6). When indicated, 1–25 ng/ml human TGF-β1 (R&D Systems) was added on days 0, 2, 4, and/or 6. On day 7, bone-marrow-derived DCs were analyzed by flow cytometry. To examine DC activation, bone-marrow-derived DCs were stimulated on day 6 with 1 or 100 ng/ml LPS from *Escherichia coli* (serotype 055:B5) (Sigma) or *Salmonella enterica* (serotype typhimurium) (Sigma) or 2∶1 ratio LPS-infected apoptotic A20 blasts [30] in the absence versus presence of TGF-β1. Sixteen hours later, cells were analyzed by flow cytometry and supernatants were analyzed by ELISA.

### Flow cytometry

Single-cell suspensions obtained from brain, spinal cord, lymph nodes, and spleen were first treated with anti-FcγIII/II receptor antibody (2-4G2) and then surface stained. Cells were stained with fluorochrome-conjugated antibodies against surface markers CD3 (500A2) (1∶100), CD4 (GK1.5) (1∶500), CD11c (HL3) (1∶150), CD11b (M1/70) (1∶100), MHCII (I-A/I-E) (M5/114) (1∶500), CD19 (1D3) (1∶500), CD45R/B220 (RA36B2) (1∶400), CD45.2 (104) (1∶200), CD49b (DX5) (1∶400), CD83 (Michel-17) (1∶100), CD86 (GL1) (1∶200), CD317/PDCA-1 (927) (1∶100), CD273/B7-DC (TY25) (1∶50), CD275/B7RP-1 (HK5.3) (1∶500), and CD357/GITR (DTA-1) (1∶100). For intracellular cytokine staining, cells were stimulated with 50 ng/ml PMA (Roche) and 500 ng/ml Ionomycin (Roche) for 5 hours at 37°C in the presence of protein-transporter inhibitor Golgi Stop (BD Biosciences). When indicated, cells were re-stimulated *in vitro* with 50 µg/ml MOG peptide for 24 hours at 37°C in the presence of protein-transporter inhibitor Golgi Stop, added during the last 5 hours of culture. Stimulated cells were first surface stained, fixed/permeabilized using CytoFix/CytoPerm kit (BD Biosciences) according to the manufacturer procedure, then stained for intracellular cytokines using fluorochrome-conjugated antibodies against IL-17 (TC11-18H10.1) (1∶500) and IFNγ (XMG1.2) (1∶200). All antibodies were purchased from BD Biosciences, eBiosciences, or Biolegend. Cells were acquired on FACSCanto or LSRII flow cytometers (BD Bioscience) using FACSDiVa software, and data were analyzed using FlowJo software (Tree Star).

### Quantitative gene expression

Total RNA was isolated with TRIzol reagent (Invitrogen) according to standard protocol. RNA was treated using DNA-free kit (Ambion) to eliminate all traces of genomic DNA. Using the standard protocol of reverse transcription, cDNA was obtained, and quantitative gene-expression analysis was conducted on an ABI Prism 7900 instrument (Applied Biosystems) using SYBR Green quantitative PCR. Primer sequence sets used in this study were obtained from Invitrogen. Data were analyzed using a 2^−ΔΔCt^ (cycle threshold) method, and results were expressed as fold of change in CD11c^dnR^ versus wild-type samples.

HPRT: Forward CTG GTG AAA AGG ACC TCT CG

Reverse TGA AGT ACT CAT TAT AGT CAA GGG CA

TGF-β1: Forward CTC CCG TGG CTT CTA GTG C

Reverse GCC TTA GTT TGG ACA GGA TCT G

IL-1: Forward ACC TGT CCT GTG TAA TGA AAG ACG

Reverse TGG GTA TTG CTT GGG ATC CA

IL-6: Forward TAG TCC TTC CTA CCC CAA TTT CC

Reverse TTG GTC CTT AGC CAC TCC TTC

IL-10: Forward GCT CTT ACT GAC TGG CAT GAG

Reverse CGC AGC TCT AGG AGC ATG TG

IL-12: Forward CAA TCA CGC TAC CTC CTC TTT T

Reverse CAG CAG TGC AGG AAT AAT GTT TC

IL-17: Forward TTT AAC TCC CTT GGC GCA AAA

Reverse CTT TCC CTC CGC ATT GAC AC

RoRγt: Forward CGA GAT GCT GTC AAG TTT GG

Reverse CAC TTG TTC CTG TTG CTG CT

IL-23: Forward ATG CTG GAT TGC AGA GCA GTA

Reverse ACG GGG CAC ATT ATT TTT AGT CT

iNos: Forward GTT CTC AGC CCA ACA ATA CAA GA

Reverse GTG GAC GGG TCG ATG TCA C

TNFα: Forward CAT CTC TTT ATC GAC GAG ACC AG

Reverse GTA TTC TTT GGA CAC GCG GTA

IFNγ: Forward ATG AAC GCT ACA CAC TGC ATC

Reverse CCA TCC TTT TGC CAG TTC CTC

### ELISA

Supernatants were collected from stimulated DCs (16 hrs), and cytokine production was measured by ELISA according to standard protocol. Capture and detection antibody pairs for IL-6 (MP5-20F3 and MP5-32C11), IL-10 (JES5-2A5 and SXC-1), and IL-12 (C15.6 and C17.8) were purchased from BD Biosciences. Recombinant mouse IL-6 (R&D), mouse IL-10 (R&D), and mouse IL-12 (Peprotech) were used as standards. Cytokine-antibody complexes were visualized by the addition of Tetramethyl Benzidine (TMB) solution (Life Technologies), and color development was stopped by the addition of TMB stop solution (Life Technologies). Absorbance at 492 nm was measured on a microplate reader (Biotek).

### Statistics

GraphPad Prism software was used to conduct unpaired, two-tailed Student's *t* tests for sample analysis. Results with *P*<0.05 were considered significant.

## Results

### CNS is the major site for TGF-β activity during EAE

**Figure 1:**
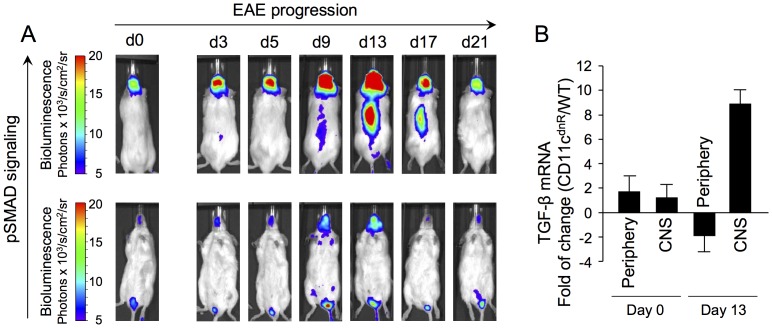
Location of TGF-β activity during EAE. (A) Bioluminescence imaging of SBE-Luc mice immunized subcutaneously with MOG peptide emulsified in CFA. Bioluminescence indicating pSMAD signaling was recorded on both the dorsal (upper panel for signal detection in the brain and spinal cord) and ventral (lower panel for signal detection in spleen and draining lymph nodes) sides of live animals at different times after EAE induction, including steady state (day 0), priming (days 3–5), pre-clinical (day 9), disease peak (day 13), and disease remission (days 17–21) phases. One representative mouse (*n* = 5) is shown. (B) SYBR Green quantitative PCR of TGF-β1 expression in the CNS and periphery of CD11c^dnR^ (*n* = 6) and wild-type (*n* = 6) mice on days 0 and 13 post-immunization. Data were analyzed using the 2^−ΔΔCt^ (cycle threshold) method, and results are expressed as the fold of change in CD11c^dnR^ versus wild-type organs. Data are representative of one (A) and two (B) independent experiments. Mean ± s.e.m. (B).

Accumulated data from *in vivo* imaging of Smad-binding-element-luciferase (SBE-luc) mice provided key information about the location of TGF-β activity during EAE [23]. These mice were engineered to express luciferase in response to Smad2/3 phosphorylation, thereby allowing the visualization of TGF-β activity in a specific and temporal manner [22]. As previously reported, we found that induction of EAE caused a prominent activity of TGF-β in the CNS, whereas no major signal was detected in the periphery (Figure 1A). Increased TGF-β activity was evident in the brain as early as day 3, followed by bioluminescence emission from the spinal cord on day 9. As EAE progressed, TGF-β activity reached maximum levels in both brain and spinal cord at the peak of disease (day 13), followed by a decline to basal levels as the disease remitted (day 21).

In sharp contrast, there was little-to-no bioluminescence emission from the periphery, indicating a demarcation of TGF-β activity in the CNS versus the periphery during EAE (Figure 1A). Such a demarcation was also established in immunized CD11c^dnR^ mice using quantitative PCR (Figure 1B). Results as expressed by the fold of change of TGF-β in CD11c^dnR^ versus wild-type organs revealed similar levels in the CNS and periphery during steady state (day 0), but higher levels in the CNS compared to the periphery at the peak of EAE (day 13). The major outcome from this study is the demonstration that the CNS, not the periphery, is the major site for TGF-β activity during EAE in CD11c^dnR^ mice. Given this finding, we re-opened the question of how a blockade of TGF-βR signaling in DCs causes severe EAE in CD11c^dnR^ mice and spontaneous EAE in CD11c^dnR^Mog^TCR^ mice [29].

### Inflamed CNS of CD11c^dnR^ mice lacking TGF-βR signaling in DCs harbors potent Th17 response

One unsolved question is whether disease severity in CD11c^dnR^ mice lacking TGF-βR signaling in DCs correlates with augmented Th17 phenotype. We have previously examined this question in the draining lymph nodes of immunized CD11c^dnR^ mice and showed substantial increase in IFNγ production when compared to immunized wild-type mice. Production of IL-17 was also increased in the lymph nodes of immunized CD11c^dnR^ mice, but to a lesser extent when compared to IFNγ production *in situ* [29]. We have now re-examined this question in the CNS because the CNS emerged as the major site for TGF-β activity during EAE (Figure 1).

**Figure 2:**
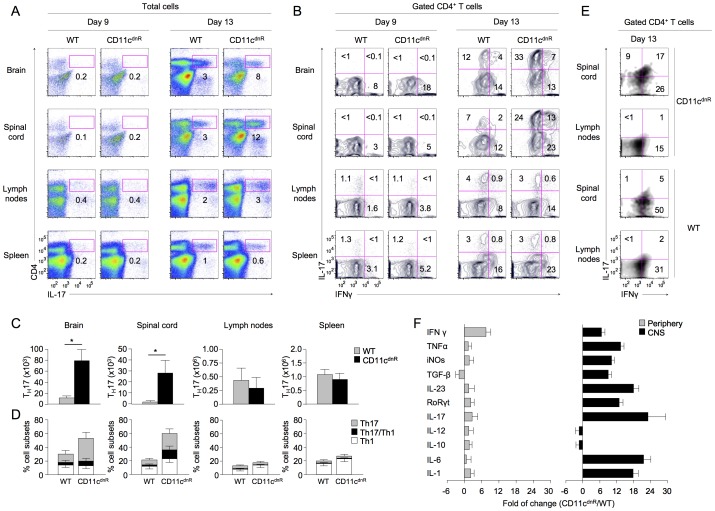
Potent Th17 differentiation uncovered in the inflamed CNS of CD11c^dnR^ mice. (A–D) Flow cytometry of CD4 versus IL-17 among total cells (A) and IL-17 versus IFNγ among gated CD4^+^ T cells (B) in brain, spinal cord, lymph nodes, and spleen of CD11c^dnR^ (*n* = 8) and wild-type (WT) (*n* = 8) mice at days 9 and 13 post-immunization. (C) Numbers of Th17 (CD4^+^IL-17^+^) cells in brain, spinal cord, lymph nodes, and spleen of CD11c^dnR^ (black) versus wild-type (WT) (gray) mice at the peak of EAE (day 13). (D) Percentages of Th17 (IL-17^+^IFNγ^−^) (gray), Th1 (IL-17^−^IFNγ^+^) (white), and Th1/Th17 (IL-17^+^IFNγ^+^) (black) cells in brain, spinal cord, lymph nodes, and spleen of CD11c^dnR^ versus wild-type (WT) mice at the peak of EAE (day 13). (E) Spinal cord and draining lymph nodes were isolated from CD11c^dnR^ (*n* = 4) and wild-type (WT) (*n* = 4) mice on day 13 post-immunization and total mononuclear cells were cultured in the presence of 50 µg/ml MOG peptide for 24 hours. Plots show the distribution of IL-17 versus IFNγ among gated CD4^+^ T cells in response to MOG re-stimulation from spinal cord versus draining lymph nodes. (F) SYBR Green quantitative PCR of the indicated genes in the CNS (black) and periphery (gray) of CD11c^dnR^ (*n* = 6) and wild-type (*n* = 6) mice at the peak of EAE (day 13). Data were analyzed using the 2^−ΔΔCt^ (cycle threshold) method, and results are expressed as the fold of change in CD11c^dnR^ versus wild-type organs. Data are representative of three (A–D) and two (E–F) independent experiments. Mean ± s.e.m. (C–D). *P<0.05 (C).

To this end, EAE was elicited in CD11c^dnR^ and wild-type mice, and outcomes on Th17 cell fate were examined in both the CNS and periphery at the peak of disease (day 13). Results, described in Figure 2, conclusively demonstrate that severe EAE in CD11c^dnR^ mice is the result of massive production of Th17 cells specifically localized at the site of CNS inflammation. First, we found that this phenotype tightly correlates with the peak of disease onset (Figure 2A and C). Second, we found that this phenotype correlates with high levels of Th17-polarizing cytokines (TGF-β, IL-1, IL-6, and IL-23) along with augmented IL-17 and its transcription factor RoRγt (Figure 2F). Third, we found that this phenotype accounts for both the conventional (IL-17^+^IFNγ^−^) and highly encephalitogenic (IL-17^+^IFNγ^+^) Th17 cell subsets (Figure 2B and D).

Notably, the frequency of IL-17^−^IFNγ^+^-producing T cells was also found to be increased in the spinal cord of immunized CD11c^dnR^ mice when compared to wild-type littermates. These IL-17^−^IFNγ^+^-producing T cells could account either for the canonical Th1 cells or for the ex-Th17 cells that have down-regulated IL-17 and up-regulated IFNγ (Figure 2B and D). Indeed, it has been shown recently that a high frequency of IFNγ-producing T cells infiltrating the CNS of MOG-immunized wild-type mice are, in fact, ex-Th17 cells. Ex-Th17 cells are by definition unstable Th17 cells that up-regulate Tbet and IFNγ and down-regulate IL-17, which leads to their conversion to ex-Th17 cells [31]–[33]. Unfortunately, ex-Th17 cells are virtually undistinguishable (phenotypically) from the canonical Th1 cells, which limit further analysis of this compartment in the diseased CD11c^dnR^ mice.

Unlike in the CNS, analysis of the periphery showed no major difference in outcomes of Th17 differentiation between CD11c^dnR^ and wild-type mice in response to EAE (Figure 2A–E). Moreover, numbers of CD4^+^ T cells in the spleen and lymph nodes of immunized mice were similar in both mouse groups, indicating similar outcomes of T-cell priming in the periphery (Figure S1A). Furthermore, analysis at the early stage of EAE (day 9) revealed similar numbers of CD4^+^ T cells infiltrating the brain and spinal cord of both mouse groups, suggesting similar rates of CNS infiltration (Figure S1B). Notably, all CD4^+^ T cells that entered the CNS on day 9 were still negative for IL-17 expression (Figure 2B). However, on day 13, significant numbers of Th17 cells were observed in the CNS of both mouse groups, but with a much greater outcome in the CNS lacking TGF-βR signaling in DCs (Figure 2E).

### Diseased CD11c^dnR^ mice lacking TGF-βR signaling in DCs exhibit highly mature DC profile in the CNS

**Figure 3:**
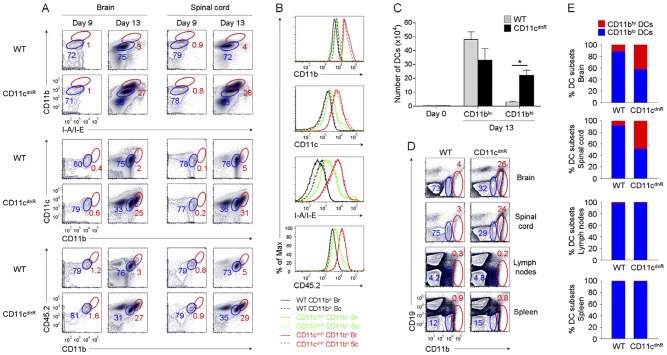
Inflamed CNS of CD11c^dnR^ mice revealed massive production of highly mature DCs lacking TGF-βR signaling. (A) Flow cytometry of CD11b versus I-A/I-E, CD11c versus CD11b, and CD45.2 versus CD11b in the brain and spinal cord of CD11c^dnR^ and wild-type (WT) mice at days 9 (*n* = 5) and 13 (*n* = 8) post-immunization. Plots are from mononuclear cells. Gates delineate two myeloid cell subsets expressing high (red) and low (blue) levels of DC maturation markers, and numbers represent the percentages of cells in red gates. (B) Overlay of gated CD11b^lo^ (black and green) and CD11b^hi^ (red) cell subsets from the brain (Br; solid) and spinal cord (Sc; dashed) of CD11c^dnR^ (green and red) and wild-type (WT) (black) mice at peak of EAE (day 13). Histograms display the fluorescence intensity of surface expression of CD11b, CD11c, I-A/I-E, and CD45.2. (C) Numbers of DC subsets (CD11b^lo^ and CD11b^hi^) recovered from the CNS of CD11c^dnR^ (black) and wild-type (WT) (gray) mice at peak of EAE (day 13). (D) Flow cytometry of CD19 versus CD11b in brain, spinal cord, lymph nodes, and spleen of CD11c^dnR^ and wild-type (WT) mice at peak of EAE (day 13). (E) Ratio of CD11b^lo^ (blue) versus CD11b^hi^ (red) DC subsets in brain, spinal cord, lymph nodes, and spleen of CD11c^dnR^ and wild-type (WT) mice at peak of EAE (day 13). Data are representative of three independent experiments. Mean ± s.e.m. (C). *P<0.05 (C).

To identify effects of TGF-β on DC lineage during EAE, we examined the profile of TGFβ-resistant DCs in the inflamed CNS, where a prominent activity of TGF-β occurs (Figure 1). To this end, EAE was elicited in CD11c^dnR^ and wild-type mice, and mononuclear cells from the brain and spinal cord were stained with myeloid DC markers (Figure 3). The goal from these experiments was to identify changes in DC profile associated with a lack of TGF-βR signaling. Strikingly, one major change emerged at the peak of disease: CNS of CD11c^dnR^ mice exhibited a distinct subset of highly mature DCs, which is otherwise suppressed in wild-type CNS (Figure 3). First, we found that this DC subset has a myeloid origin, as indicated by high levels of CD11b and CD11c in the inflamed CNS of CD11c^dnR^ mice (Figures 3A and B). Second, strong up-regulation of MHC class II on these DCs suggested high levels of maturation and potent antigen presentation in the inflamed CNS of CD11c^dnR^ mice (Figures 3A and B). Third, high levels of CD45.2 indicated that this DC subset is not a CNS-resident cell type, but rather a hematopoietic-derived cell type (Figure 3A and B).

A DC subset with such a profile (CD45.2^hi^CD11b^hi^CD11c^hi^MHCII^hi^) was found abundantly in the inflamed CNS of CD11c^dnR^ mice, whereas much fewer were detected in the inflamed CNS of wild-type littermates (Figure 3C). Importantly, these DCs were absent from the healthy CNS (day 0) (Figure 3C) and were undetectable before the disease onset (day 9) (Figure 3A). They were uncovered by the lack of TGF-βR signaling at the peak of EAE (day 13), a phenotype that strongly correlates with high levels of inflammation (Figure 2F) and potent Th17 cell differentiation (Figure 2A–E).

### Numbers of TGF-β-resistant DCs at the site of inflammation correlate with gain in Th17 differentiation *in situ*

**Figure 4:**
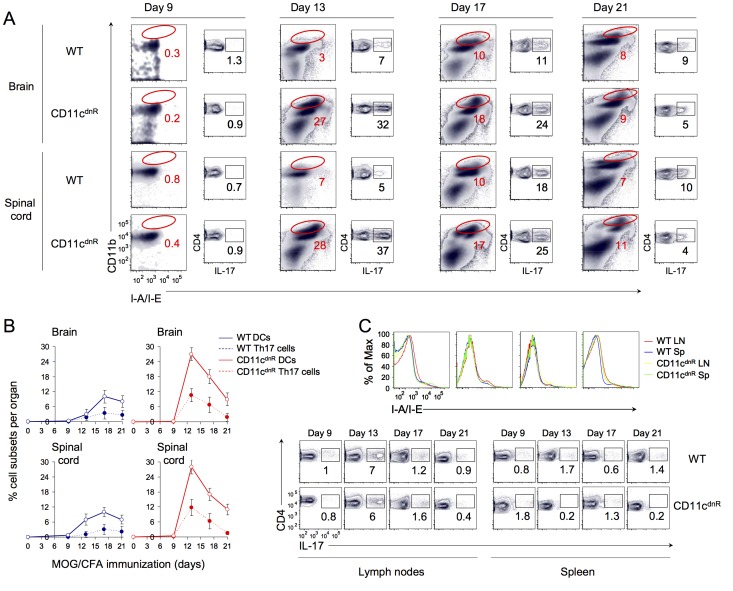
Kinetics of mature DC production lacking TGF-βR signaling correlate with augmented Th17 differentiation in the inflamed CNS. (A) Flow cytometry of CD11b versus I-A/I-E from total cells and CD4 versus IL-17 from gated CD4^+^T cells in the brain and spinal cord of CD11c^dnR^ (*n* = 3) and wild-type (WT) (*n* = 3) mice at days 9, 13, 17, and 21 post-immunization. Numbers indicate the frequency of CD11b^hi^MHC^hi^ DCs and Th17 cells, respectively. (B) Kinetics of percentages of CD11b^hi^MHC^hi^ DCs (solid line) and Th17 cells (dashed line) in brain and spinal cord from CD11c^dnR^ (red) and wild-type (WT) (blue) mice at days 9, 13, 17, and 21 post-immunization. (C) Histograms display the fluorescence intensity of surface expression of I-A/I-E from B-cell negative gate in lymph nodes (red and yellow) and spleen (blue and green) of CD11c^dnR^ (yellow and green) and wild-type (WT) (red and blue) mice at days 9, 13, 17, and 21 post-immunization. Plots show the distribution CD4 versus IL-17 from gated CD4^+^ T cells in the lymph nodes and spleen of CD11c^dnR^ and wild-type (WT) mice at days 9, 13, 17, and 21 post-immunization. Data are representative of two independent experiments. Mean ± s.e.m. (B).

High numbers of CD45.2^hi^CD11b^hi^CD11c^hi^MHCII^hi^ DCs in the inflamed CNS of CD11c^dnR^ mice raised a central question: Is their differentiation/production specific to the inflamed CNS or does it originate in the priming site? To address this question, EAE was elicited in CD11c^dnR^ and wild-type mice, and distribution of this DC phenotype was examined in the CNS versus the periphery (Figure 3D and E). Interestingly, while CD45.2^hi^CD11b^hi^CD11c^hi^MHCII^hi^ DCs were abundant in the CNS of immunized CD11c^dnR^, this phenotype was not detected in the spleens or lymph nodes (Figure 3D and E). To better define the relationship between levels of TGF-β activity in the inflamed CNS and numbers of CD45.2^hi^CD11b^hi^CD11c^hi^MHCII^hi^ DCs, we traced this DC phenotype as TGF-β activity peaks (day 13) and declines (day 21) in the CNS during the course of EAE (Figure 4). Results, as indicated by kinetics of wild-type DC numbers in the brain and spinal cord, revealed an inverse correlation between levels of TGF-β activity in the inflamed CNS and numbers of CD45.2^hi^CD11b^hi^CD11c^hi^MHCII^hi^ DCs *in situ* (Figure 4A and B).

Importantly, numbers of CD45.2^hi^CD11b^hi^CD11c^hi^MHCII^hi^ DCs in the inflamed CNS were proportionally mirrored by numbers of Th17 cells recovered *in situ* (Figure 4A and B). Notably, no difference was observed in the peripheral DC compartment between CD11c^dnR^ and wild-type mice during the course of EAE, and this was mirrored by similar outcomes of Th17 differentiation in the periphery of both mouse groups (Figure 4C). To further extend this observation, we next characterized the peripheral DC compartment lacking TGF-βR signaling in a steady-state condition (Figure S2). Results, as indicated by the expression of MHC class II, CD11c, CD8, PDCA-1, B220, CD83, CD86, B7-DC, B7-rp1, and GITR, revealed no difference in healthy CD11c^dnR^ mice compared to wild-type littermates.

Finally, because NK cells from CD11c^dnR^ mice lack TGF-βR signaling, we asked whether they have, like TGF-β-resistant DCs, different behavior in the inflamed versus the priming site (Figure S3). However, unlike TGF-β-resistant DCs, we found that TGF-β-resistant NK cells have a similar profile in the inflamed CNS versus the periphery in response to EAE. This finding is in line with our previous work showing that severe EAE in CD11c^dnR^ mice is the result of lack of TGF-βR signaling in DCs but not in NK cells [29]. Together, our data suggest a specific role for the interplay between TGF-β and DCs at the site of inflammation but not at the priming site.

### Highly numbers of mature DCs lacking TGF-βR is a common denominator of disease severity

**Figure 5:**
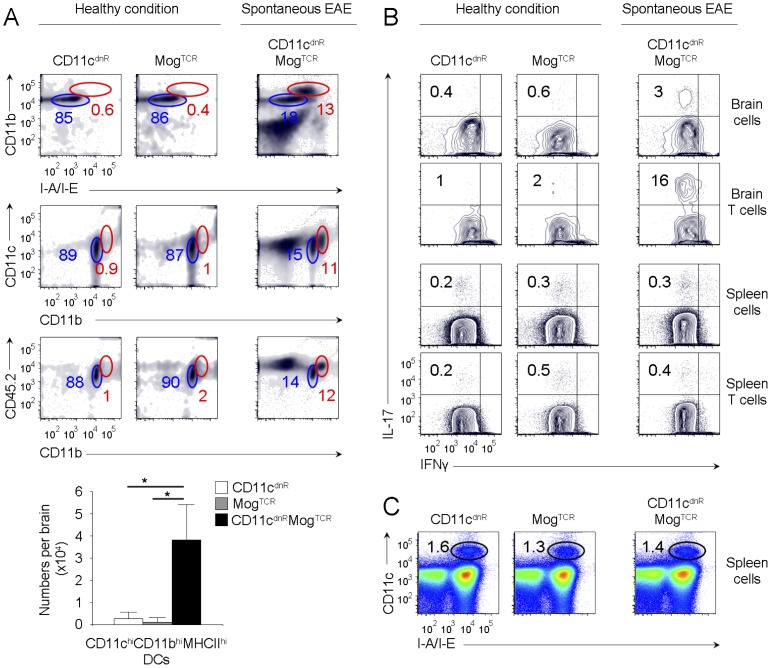
Disease severity in CD11c^dnR^Mog^TCR^ mice correlates with CNS uncontrolled production of mature DCs lacking TGF-βR signaling. (A) Flow cytometry of CD11b versus I-A/I-E, CD11c versus CD11b, and CD45.2 versus CD11b in the brain of untreated CD11c^dnR^ (*n* = 6), Mog^TCR^ (*n* = 3), and CD11c^dnR^Mog^TCR^ (*n* = 6) mice. Plots are from mononuclear cells. Gates delineate two myeloid cell subsets expressing high (red) and low (blue) levels of DC maturation markers, and numbers represent the percentages of cells in red gates. Bar graphs summarize the number of CD45.2^hi^CD11b^hi^CD11c^hi^MHCII^hi^ DCs (as identified by red gates) recovered from the brain of untreated CD11c^dnR^ (white), Mog^TCR^ (gray), and CD11c^dnR^Mog^TCR^ (black) mice. (B) Flow cytometry of IL-17 versus IFNγ is shown in total mononuclear cells as well as among gated CD4^+^ T cells in the brain and spleen of untreated CD11c^dnR^ (*n* = 3), Mog^TCR^ (*n* = 3), and CD11c^dnR^Mog^TCR^ (*n* = 3) mice. Numbers in quadrants indicate the frequency of Th17 cells. (C) Flow cytometry of CD11c versus I-A/I-E in the spleen of untreated CD11c^dnR^ (*n* = 6), Mog^TCR^ (*n* = 3), and CD11c^dnR^Mog^TCR^ (*n* = 6) mice. Data are representative of three (A, C) and two (B) independent experiments. Mean ± s.e.m. (A). *P<0.05 (A).

CD11c^dnR^Mog^TCR^ mice lacking TGF-βR signaling in DCs provide an excellent model of spontaneous EAE-like disease previously described by CNS inflammation, infiltration of activated T cells in the CNS, impaired locomotion, and premature death [29]. Thus, to ascertain results above, we next examined DC phenotype in CD11c^dnR^Mog^TCR^ mice suffering from spontaneous EAE (Figure 5A and C). We found substantial numbers of mature myeloid DC type in the CNS of CD11c^dnR^Mog^TCR^ mice suffering from spontaneous EAE, whereas this phenotype was undetectable in healthy CNS (Figure 5A). Importantly, high numbers of mature DCs in the CNS of CD11c^dnR^Mog^TCR^ mice with spontaneous EAE correlated with Th17 cell differentiation *in situ* (Figure 5B) Notably, neither CD45.2^hi^CD11b^hi^CD11c^hi^MHCII^hi^ DCs nor Th17 cells were detected in the periphery of CD11c^dnR^Mog^TCR^ mice with spontaneous EAE (Figure 5B and C). Thus, the common denominator between severe EAE in immunized CD11c^dnR^ mice and spontaneous EAE in crossed CD11c^dnR^Mog^TCR^ mice is the differentiation of highly mature DCs lacking TGF-βR signaling in the CNS that correlates with potent Th17 differentiation *in situ*.

### TGF-β blocks DC formation but not DC activation

**Figure 6:**
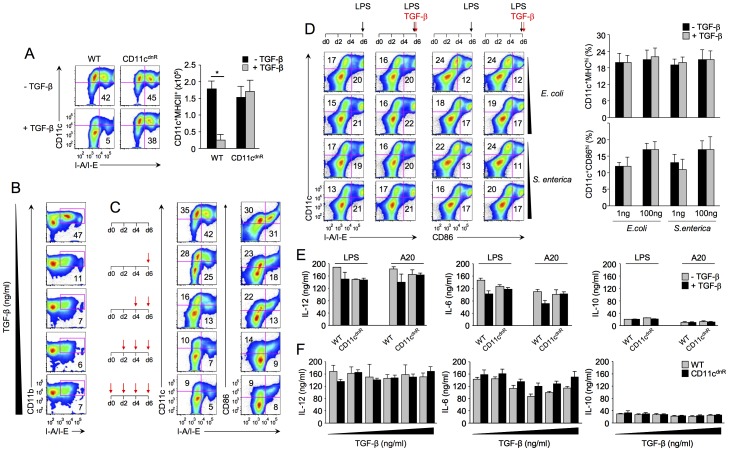
TGF-β suppresses DC production but has no effect on DC activation. (A) Flow cytometry of DCs derived from bone-marrow precursors for 6 days in the absence or presence of 5 ng/ml TGF-β. Plots show the distribution of CD11c versus I-A/I-E, and numbers in quadrants indicate the percentage of CD11c^+^MHCII^+^ DCs. Bar graphs indicate the number of CD11c^+^MHCII^+^ DCs derived from CD11c^dnR^ (*n* = 6) and wild-type (WT) (*n* = 6) cultures in the absence (black) versus presence (gray) of TGF-β. (B) Flow cytometry of DCs derived from wild-type bone-marrow precursors (*n* = 4) cultured for 6 days in the presence of different doses of TGF-β (0, 1, 5, 10, and 25 ng/ml). Plots show the distribution of CD11b versus I-A/I-E, and numbers in quadrants indicate the percentage of CD11b^+^MHCII^+^ DCs. (C) Flow cytometry of DCs from wild-type bone-marrow precursors cultured in the presence of TGF-β at different times and for different durations as indicated by red arrows (each red arrow indicates the addition of TGF-β). Plots show the distribution of CD11c versus I-A/I-E and CD86 versus I-A/I-E in each combination. (D) Flow cytometry of bone marrow derived DCs stimulated with LPS (black arrow) for 16 hrs in the absence or presence of TGF-β (red arrow). Plots show the distribution of CD11c versus I-A/I-E and CD11c versus CD86 from wild-type cultures (*n* = 4). Bar graphs summarize the average frequency of DCs in response to two doses (1 and 100 ng) and two sources (*E. coli* and *S. enterica*) of LPS stimulation in the absence (black) or presence (gray) of 5 ng/ml TGF-β. (E-F) Cytokine production measured by ELISA in the supernatants collected from bone-marrow-derived DCs stimulated with soluble LPS (E–F) or LPS-A20 (E) for 16 hrs in the absence or presence of TGF-β at 5 ng/ml (E) or TGF-β at 1, 5, 10, 25, and 100 ng/ml (F). Bar graphs summarize the average production of IL-12, IL-6, and IL-10 from CD11c^dnR^ (*n* = 4) and wild-type (WT) (*n* = 4) cultures. Data are representative of four (A), two (B, C), and three (E, F) independent experiments. Mean ± s.e.m. (A and D–F). *P<0.05 (A).

One important question emerged from results above: Does abundant TGF-β in the neuroinflammation site suppress DCs at a precursor level or at mature stage? The generation of DCs can be modeled *in vitro* by bone-marrow-derived DCs cultured with GM-CSF [34], [35]. Thus, to address this question, we used this approach and directly assessed effects of TGF-β on DC formation versus activation *in vitro* (Figure 6).

In the first set of experiments, we examined outcomes of DC formation in the absence versus presence of TGF-β (Figure 6A–C). In the absence of TGF-β, we found that bone-marrow precursors from CD11c^dnR^ and wild-type mice were equally efficient in generating DCs. In sharp contrast, the addition of TGF-β severely blocked the development of wild-type DCs, but had no major effects on TGF-β-resistant DCs, as expected (Figure 6A). Such a blockade is well demonstrated by the aborted expression of CD11c, CD11b, CD86, and MHC class II in wild-type cultures supplemented with TGF-β (Figure 6A–C). Notably, 1 ng/ml of TGF-β was sufficient to block wild-type DC formation (Figure 6B). To trace the effects of TGF-β along the DC derivation pathway, cultures were supplemented with TGF-β at different time points and for different durations. On day 7, cultures were examined for DC formation, as shown in Figure 6C and summarized in Figure S4. Results from 16 on/off combinations of TGF-β revealed that TGF-β is most potent in suppressing DC production at a precursor level.

In the second set of experiments, bone-marrow-derived DCs were stimulated with bacterial products and effects of TGF-β on TLR-induced activation were assessed at both surface-expression and cytokine-production levels (Figure 6D and F). Strikingly, in contrast to DC formation, the addition of TGF-β had no effect on TLR-induced activation (Figure 6D and F). Specifically, levels of CD11c, CD86, and MHC class II remained unchanged in the presence of TGF-β, and this finding was confirmed using LPS from two sources (*Escherichia coli* and *Salmonella enterica*) and two doses (1 and 100 ng/ml) (Figure 6D). Similarly, we found that production of IL-12, IL-10, and IL-6 did not differ between wild-type and TGF-β-resistant DCs stimulated with LPS at different times (Figure S5), LPS as compared to LPS-infected apoptotic cells (Figure 6E), or LPS in the absence or presence of TGF-β at different doses (Figure 6F). Collectively, our data identify TGF-β as a negative regulator of DC generation but not DC activation. This suggests an unprecedented demarcation of TGF-β activity in DC formation versus DC activation.

## Discussion

A chief pursuit in the field of T-cell differentiation is deciphering the factors behind the ‘decision’ to mount or block a T cell lineage. Among these factors, TGF-β emerged as a master regulator behind this decision, proved by its ability to block Th1 and Th2 cells while promoting Th17 cell differentiation [4], [5], [8]–[10]. These conclusions are largely based on the results of T-cell stimulation *in vitro*, where TGF-β actions are assessed in a T-cell intrinsic pathway. While such a reductionist strategy is remarkably powerful in revealing how TGF-β regulates T cell differentiation, it is limited to recapitulate the complex interaction between TGF-β and the various immune cell types involved in the physiological context of autoimmunity or inflammation. This study suggests that TGF-β can restrict Th17 differentiation via DCs specifically at the site of inflammation.

DCs are increasingly recognized as a highly plastic continuum of cells that can adopt different functions, including regulatory and inflammatory roles, depending on their location and the physiological context [13]. Accumulating evidence indicates that the microenvironment established in the CNS during autoimmune inflammation is conducive to development of pro-inflammatory DCs capable of driving Th17 cell differentiation [14], [36], [37]. However, how these CNS DCs are regulated remains poorly understood. In this study, we provide evidence that TGF-β regulates DCs in the inflamed CNS. EAE development in mice carrying a DC-specific blockade of TGF-βR signaling uncovered high numbers of mature DCs in the inflamed CNS that are otherwise suppressed in the CNS of wild-type controls. These TGF-β-resistant DCs expressed high levels of CD11b and CD11c, indicating a myeloid origin; strong up-regulation of MHC class II, indicating high levels of maturation and potent antigen presentation; and high levels of CD45.2, indicating hematopoietic derivation. Importantly, their detection was confined with the site of neuroinflammation and correlated with potent Th17 differentiation. While it is undisputable that the TGF-β intrinsic pathway drives Th17 differentiation, our data suggest that Th17 cell fate is shaped by TGF-β extrinsic pathway via DCs.

The exact role of TGF-β in DCs has remained a puzzling question since the discovery that CD11c^dnR^ mice do not display impaired DC development and homeostasis at steady state, yet a lack of TGF-βR signaling in DCs proved to be responsible for severe EAE [29], [38]. By comparing CNS and periphery in immunized CD11c^dnR^ mice (suffering from severe EAE) and crossed CD11c^dnR^Mog^TCR^ mice (suffering from spontaneous EAE), our data revealed a role for TGF-β in DCs at the inflammatory (CNS) but not priming (periphery) sites. Such a demarcation is supported by abundant numbers of highly mature DCs in the inflammatory (CNS) but not in the priming (periphery) sites of diseased CD11c^dnR^ mice. In the periphery, results showed no difference between wild-type and TGF-β-resistant DCs, and this was confirmed in healthy as well as in diseased conditions.

A unifying explanation for this dichotomy is provided by the selective location of TGF-β activity in the inflamed CNS. In this environment, several scenarios could explain the phenotype of abundant mature TGFβ-resistant DCs in the inflamed CNS. One possibility is enhanced recruitment of monocytes which then differentiate into highly mature DCs due to the existing pro-inflammatory environment. Abundant Th17 cells in the CNS of CD11c^dnR^ mice could lead to more GM-CSF and hence more DC recruitment and differentiation which in return will lead to more Th17 cells either through activation and expansion of already committed T cells or through de novo differentiation of Th17 cells in situ. It is also possible that high numbers of mature DCs in the CNS of CD11c^dnR^ mice is the result of enhanced in situ differentiation of DCs at a precursor level.

Previous studies on the lack of TGF-βR signaling in NK cells placed suppressive effects of TGF-β at both precursor and mature levels, as shown by the (i) regulation of the terminal step of NK-cell development in the bone marrow [39], and (ii) inhibition of mature NK-cell production of IFNγ [38]. In T cells, however, results converged to show opposing effects of TGF-β, as indicated by the (i) regulation of iNKT-cell development in the thymus [40], (ii) inhibition of T-cell proliferation and IL-2 production [2], [3], (iii) suppression of Th1 and Th2 differentiation [4], [5], and (iv) promotion of Treg and Th17 differentiation [6], [8]–[10]. Unlike lymphocytes, our data showed that TGF-β in DCs has effects on DC formation but not DC activation *in vitro*. Specifically, we found that TGF-β is a potent suppressor of DC derivation by GM-CSF while completely ineffective on DC activation by LPS.

Our data are in line with recent studies showing that during the interplay between LPS and TGF-β signals, LPS overrides and suppresses TGF-β signaling [41]. In addition, the fact that TGF-β does not suppress DC at a mature level provides an essential regulatory mechanism that allows TGF-β to drive naive T cells into Th17 differentiation without depressing the DC function required for this process. Finally, the integration of our results obtained from both *in vivo* and *in vitro* studies provides us with a model in which abundant TGF-β in the neuroinflammatory site could be responsible for blocking *de novo* generation of CD45.2^hi^CD11b^hi^CD11c^hi^MHCII^hi^ DCs that might be programmed in this particular milieu to promote Th17 differentiation. Although it is tempting to propose that this critical control by TGF-β targets the formation of inflammatory DC type, future studies are required to investigate this possibility.

In summary, while extensive efforts have been devoted to identifying the precursor cell that generates DCs and the soluble factor that mediates their differentiation [13], our study provides insight into the mechanism of their suppression. Our finding that TGF-β is the factor responsible for controlling the formation of CD45.2^hi^CD11b^hi^CD11c^hi^MHCII^hi^ DCs in the neuroinflammatory site holds unprecedented implications for our understanding of Th17-cell differentiation in the physiological context of inflammation and provides a foundation for the pursuit of tailored DC-based therapies against autoimmune inflammatory diseases.

## Supporting Information

Figure S1 Similar numbers of CD4^+^ T cells infiltrate the CNS of CD11c^dnR^ and wild type mice during the early phase of EAE. Flow cytometry of CD4 versus CD3 in lymph nodes and spleen (A) and brain and spinal cord (B) isolated from CD11c^dnR^ (*n* = 3) and wild-type (WT) (*n* = 3) mice at days 9 and 13 post-immunization. Numbers indicate percentages of cells in gate. Bar graphs summarize the average frequency of CD4^+^ T cells in CD11c^dnR^ (black) versus wild-type (WT) (gray) organs. Data are representative of three independent experiments. Mean ± s.e.m.
(TIFF)

Figure S2 Lack of TGF-βR signaling has no effect on DC development at steady-state. (A, B) Flow cytometry of cells isolated from lymph nodes, spleen, and bone marrow of CD11c^dnR^ (*n* = 3) and wild-type (WT) (*n* = 3) mice at steady state. (A) Plots show the distribution of I-E/I-A versus CD11c, CD8, PDCA1, B220, CD83, CD86, B7-DC, B7rp1, and GITR in each organ. (B) I-E/I-A versus DX5 staining is shown as control indicating higher frequency of NK cells in CD11c^dnR^ compared to wild type (WT) mice. Numbers indicate percentages of cells in gates and data are representative of two independent experiments.
(TIFF)

Figure S3 NK cells lacking TGF-βR signaling display similar profile in CNS versus periphery in response to EAE. (A) Flow cytometry of NK1.1 versus CD3 in brain, spinal cord, lymph nodes, and spleen isolated from immunized CD11c^dnR^ (*n* = 4) and wild-type (WT) (*n* = 4) mice at peak of EAE (day 13). Bar graphs summarize the average frequency of NK cells in CD11c^dnR^ (black) versus wild-type (WT) (gray) organs. (B) Brain, spinal cord, lymph nodes, and spleen were isolated from immunized CD11c^dnR^ and wild-type (WT) mice at peak of EAE (day 13). Plots show the distribution of NK1.1 versus IFNγ after 4 hours of re-stimulation with a combination of IL-12 (10 ng/ml) and IL-18 (20 ng/ml). Bar graphs summarize the average frequency of IFNγ-expressing NK cells in CD11c^dnR^ (black) versus wild-type (WT) (gray) organs. Data are representative of three independent experiments. Mean ± s.e.m.
(TIFF)

Figure S4 Suppressive effects of TGF-β wanes as DC mature. Bone marrow precursors were cultured with GM-CSF for 6 days in the absence or presence of 5 ng/ml TGF-β at different times and for different durations as indicated by red arrows (each red arrow indicates the addition of TGF-β). Bar graphs summarize the average frequency of wild type DCs (*n* = 6) recovered from 16 on/off combinations of TGF-β. The frequency of CD11c^+^MHCII^+^ DCs is expressed as a percentage relative to the condition without TGF-β. Data are representative of three independent experiments. Mean ± s.e.m.
(TIFF)

Figure S5 DCs derived from CD11c^dnR^ and wild type precursors are equally potent in cytokine production. Cytokine production measured by ELISA in supernatants from bone marrow derived DCs in response to LPS stimulation (100 ng/ml). Bar graphs summarize the average production of IL-12, IL-6, and IL-10 from CD11c^dnR^ (*n* = 3) and wild-type (WT) (*n* = 3) DCs stimulated with LPS for 6 (white), 12 (grey), and 18 (black) hours. Data are representative of three independent experiments. Mean ± s.e.m.
(TIFF)
