# Supplementary figures and images for: TGF-β Signaling Initiated in Dendritic Cells Instructs Suppressive Effects on Th17 Differentiation at the Site of Neuroinflammation
Source: PLoS One. 2014 Jul 29;9(7):e102390. doi: 10.1371/journal.pone.0102390 (PMC4114567; doi:10.1371/journal.pone.0102390)

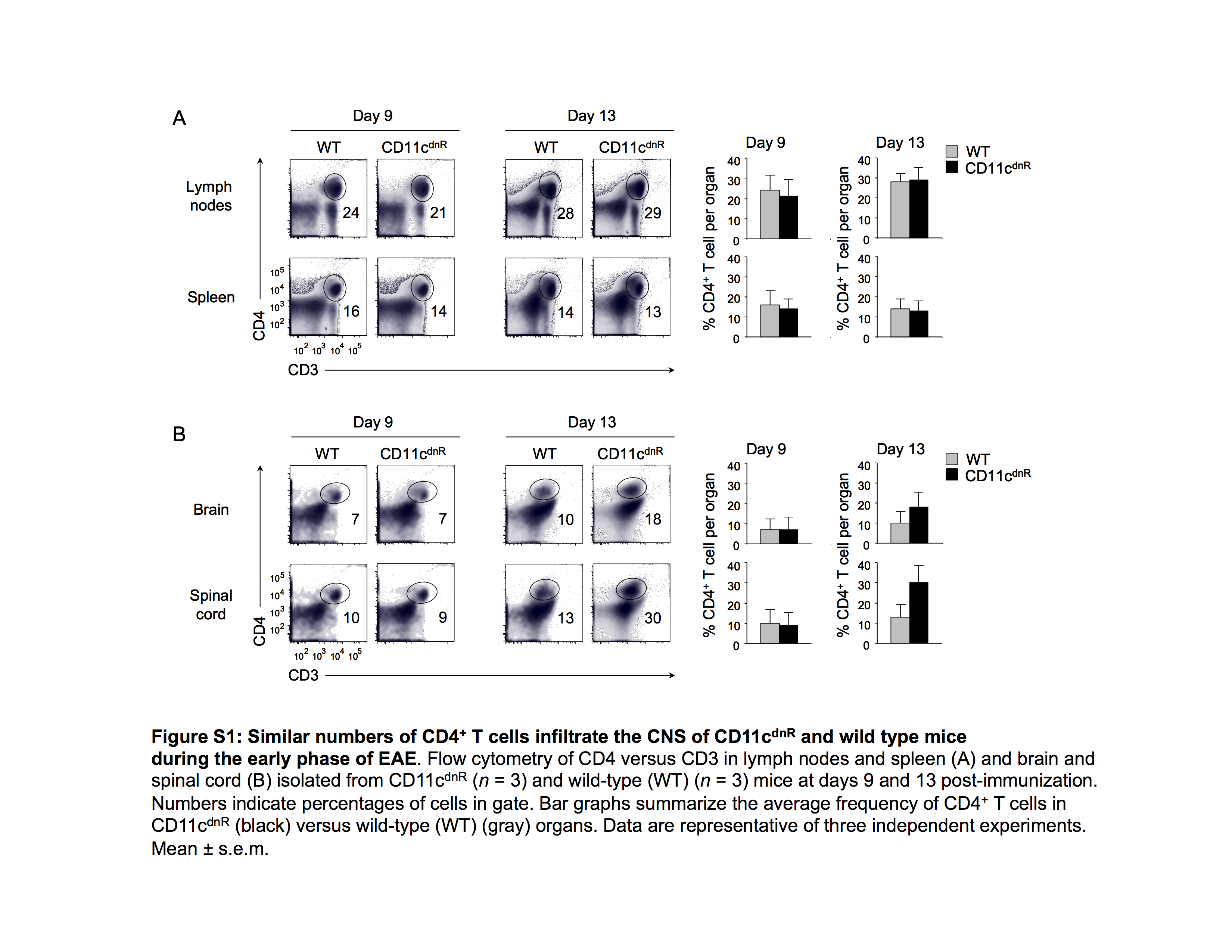

Supplement: Figure S1 — Similar numbers of CD4+ T cells infiltrate the CNS of CD11cdnR and wild type mice during the early phase of EAE. Flow cytometry of CD4 versus CD3 in lymph nodes and spleen (A) and brain and spinal cord (B) isolated from CD11cdnR (n = 3) and wild-type (WT) (n = 3) mice at days 9 and 13 post-immunization. Numbers indicate percentages of cells in gate. Bar graphs summarize the average frequency of CD4+ T cells in CD11cdnR (black) versus wild-type (WT) (gray) organs. Data are representative of three independent experiments. Mean ± s.e.m. (TIFF) [file pone.0102390.s001.tiff]

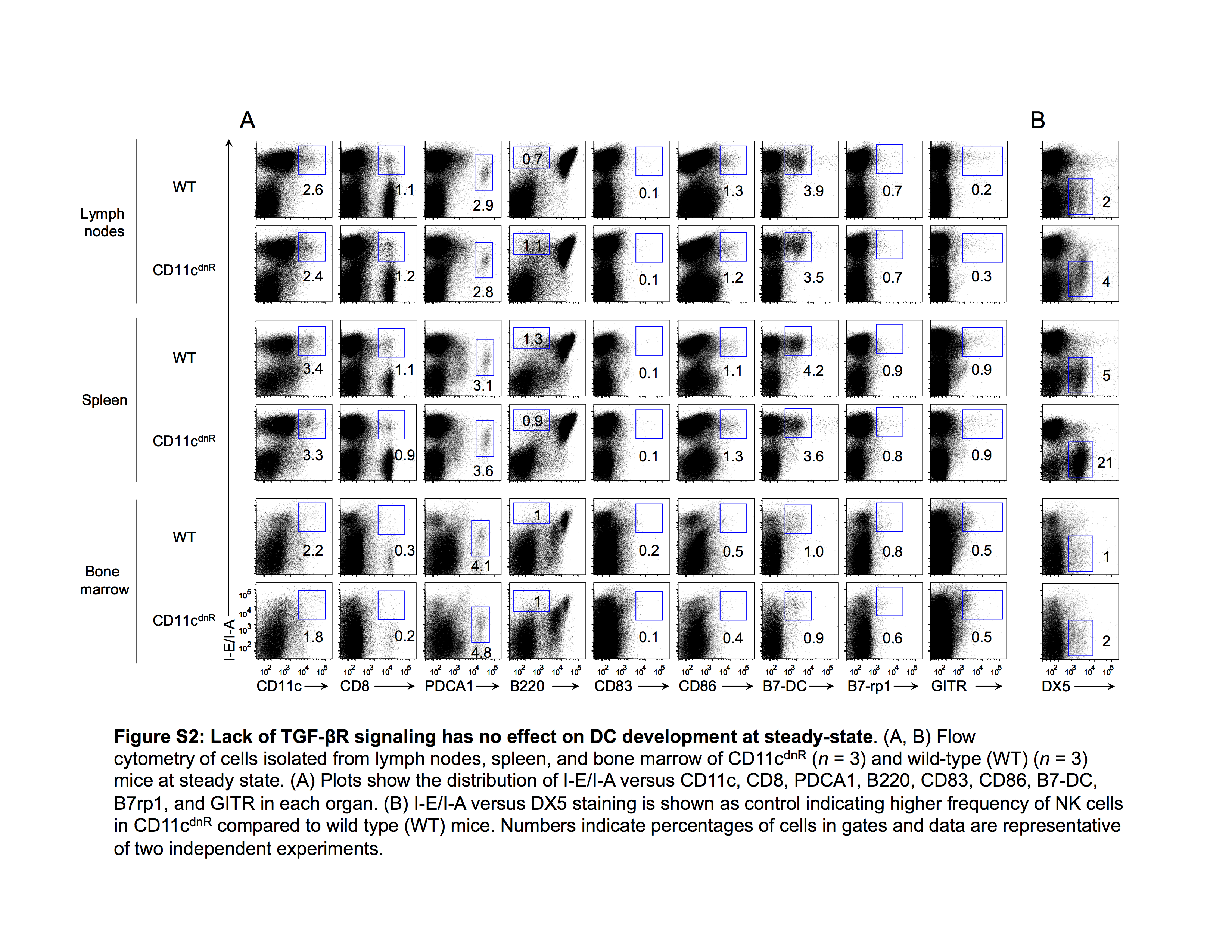

Supplement: Figure S2 — Lack of TGF-βR signaling has no effect on DC development at steady-state. (A, B) Flow cytometry of cells isolated from lymph nodes, spleen, and bone marrow of CD11cdnR (n = 3) and wild-type (WT) (n = 3) mice at steady state. (A) Plots show the distribution of I-E/I-A versus CD11c, CD8, PDCA1, B220, CD83, CD86, B7-DC, B7rp1, and GITR in each organ. (B) I-E/I-A versus DX5 staining is shown as control indicating higher frequency of NK cells in CD11cdnR compared to wild type (WT) mice. Numbers indicate percentages of cells in gates and data are representative of two independent experiments. (TIFF) [file pone.0102390.s002.tiff]

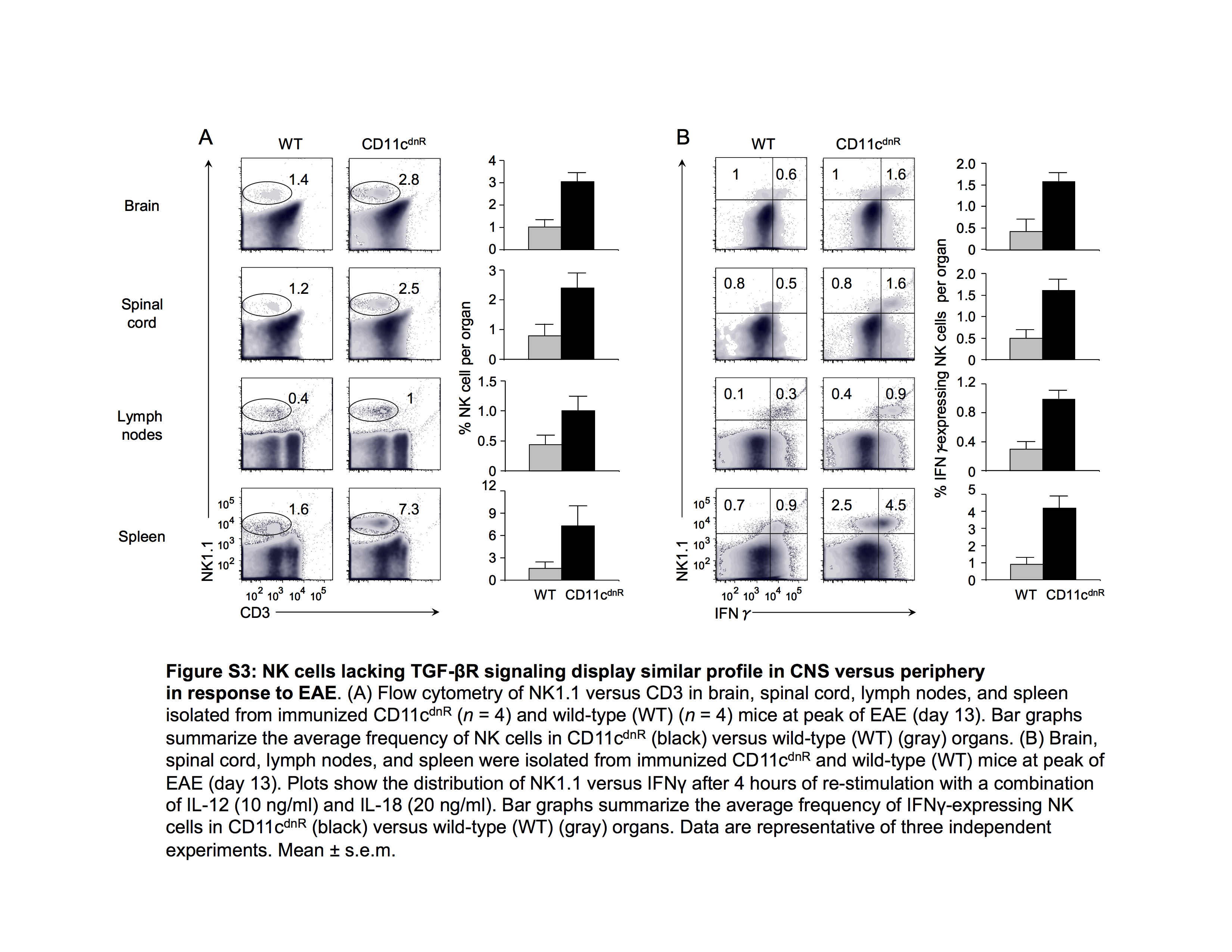

Supplement: Figure S3 — NK cells lacking TGF-βR signaling display similar profile in CNS versus periphery in response to EAE. (A) Flow cytometry of NK1.1 versus CD3 in brain, spinal cord, lymph nodes, and spleen isolated from immunized CD11cdnR (n = 4) and wild-type (WT) (n = 4) mice at peak of EAE (day 13). Bar graphs summarize the average frequency of NK cells in CD11cdnR (black) versus wild-type (WT) (gray) organs. (B) Brain, spinal cord, lymph nodes, and spleen were isolated from immunized CD11cdnR and wild-type (WT) mice at peak of EAE (day 13). Plots show the distribution of NK1.1 versus IFNγ after 4 hours of re-stimulation with a combination of IL-12 (10 ng/ml) and IL-18 (20 ng/ml). Bar graphs summarize the average frequency of IFNγ-expressing NK cells in CD11cdnR (black) versus wild-type (WT) (gray) organs. Data are representative of three independent experiments. Mean ± s.e.m. (TIFF) [file pone.0102390.s003.tiff]

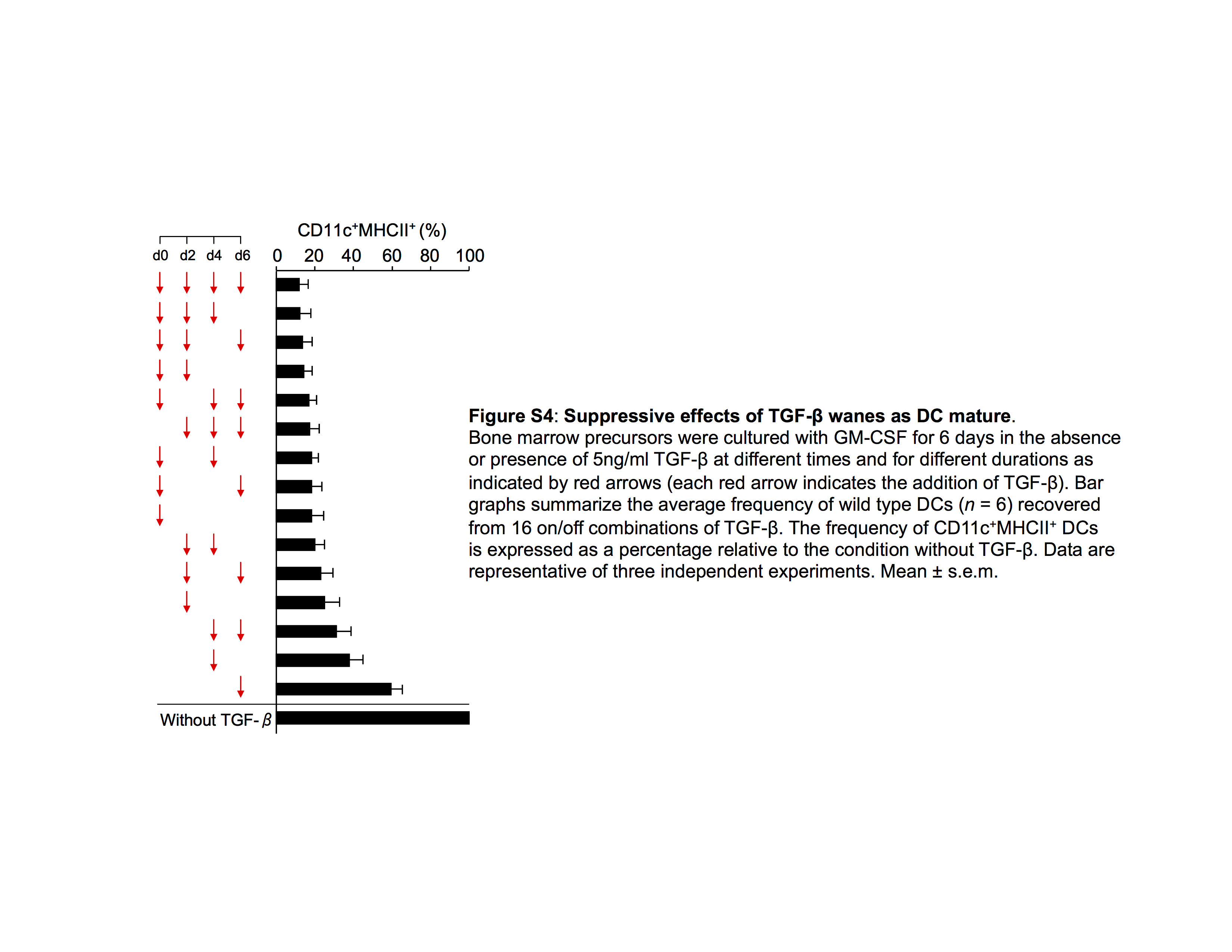

Supplement: Figure S4 — Suppressive effects of TGF-β wanes as DC mature. Bone marrow precursors were cultured with GM-CSF for 6 days in the absence or presence of 5 ng/ml TGF-β at different times and for different durations as indicated by red arrows (each red arrow indicates the addition of TGF-β). Bar graphs summarize the average frequency of wild type DCs (n = 6) recovered from 16 on/off combinations of TGF-β. The frequency of CD11c+MHCII+ DCs is expressed as a percentage relative to the condition without TGF-β. Data are representative of three independent experiments. Mean ± s.e.m. (TIFF) [file pone.0102390.s004.tiff]

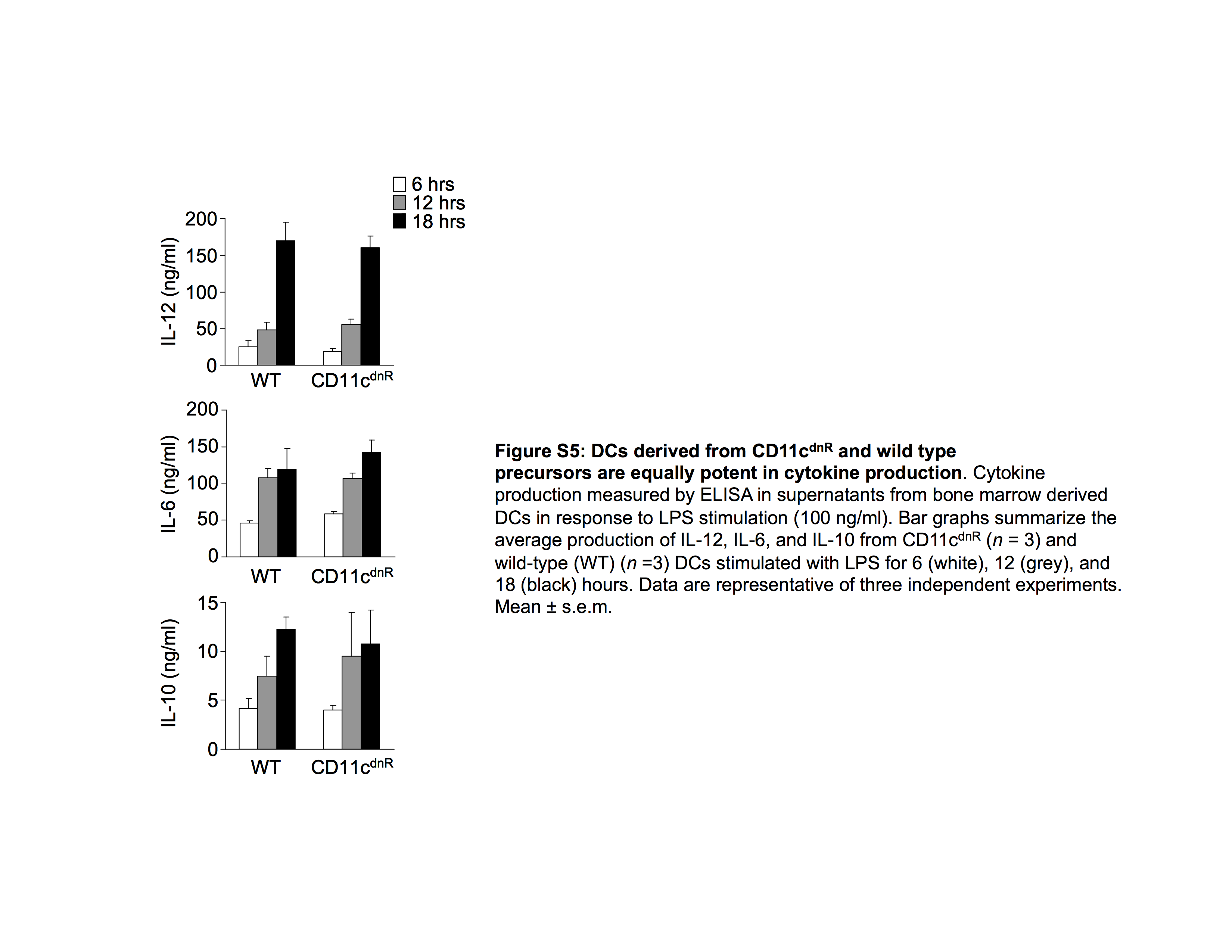

Supplement: Figure S5 — DCs derived from CD11cdnR and wild type precursors are equally potent in cytokine production. Cytokine production measured by ELISA in supernatants from bone marrow derived DCs in response to LPS stimulation (100 ng/ml). Bar graphs summarize the average production of IL-12, IL-6, and IL-10 from CD11cdnR (n = 3) and wild-type (WT) (n = 3) DCs stimulated with LPS for 6 (white), 12 (grey), and 18 (black) hours. Data are representative of three independent experiments. Mean ± s.e.m. (TIFF) [file pone.0102390.s005.tiff]
